# The Identification of RNA Modification Gene *PUS7* as a Potential Biomarker of Ovarian Cancer

**DOI:** 10.3390/biology10111130

**Published:** 2021-11-03

**Authors:** Huimin Li, Lin Chen, Yunsong Han, Fangfang Zhang, Yanyan Wang, Yali Han, Yange Wang, Qiang Wang, Xiangqian Guo

**Affiliations:** 1 Cell Signal Transduction Laboratory, Bioinformatics Center, Henan Provincial Engineering Center for Tumor Molecular Medicine, Institute of Biomedical Informatics, School of Basic Medical Sciences, Henan University, Kaifeng 475001, China; lihuimin0202@163.com (H.L.); cyy050067@163.com (L.C.); 18298352872@163.com (Y.H.); zff11211281010@163.com (F.Z.); w18237833015@163.com (Y.W.); hangreen5508@163.com (Y.H.); wangyange3@henu.edu.cn (Y.W.); 2 School of Software, Henan University, Kaifeng 475001, China

**Keywords:** DEGs, diagnosis, ovarian cancer, PUS7, RMGs

## Abstract

**Simple Summary:**

RNA modifications are involved in a variety of diseases, including cancers. Given the lack of efficient and reliable biomarkers for early diagnosis of ovarian cancer (OV), this study was designed to explore the role of RNA modification genes (RMGs) in the diagnosis of OV. The study first selected PUS7 (Pseudouridine Synthase 7) as a diagnostic biomarker candidate through the analysis of differentially expressed genes using TCGA and GEO data. Then, we evaluated its specificity and sensitivity using Receiver Operating Characteristic (ROC) analysis in TCGA and GEO data. The protein expression, mutation, protein interaction networks, correlated genes, related pathways, biological processes, cell components, and molecular functions were analyzed for PUS7 as well. The upregulation of PUS7 protein in OV was confirmed by the staining images in HPA and tissue arrays. In conclusion, the findings of the present study point towards the potential of PUS7 as the diagnostic marker and therapeutic target for ovarian cancer.

**Abstract:**

RNA modifications are reversible, dynamically regulated, and involved in a variety of diseases such as cancers. Given the lack of efficient and reliable biomarkers for early diagnosis of ovarian cancer (OV), this study was designed to explore the role of RNA modification genes (RMGs) in the diagnosis of OV. Herein, 132 RMGs were retrieved in PubMed, 638 OV and 18 normal ovary samples were retrieved in The Cancer Genome Atlas (TCGA), and GSE18520 cohorts were collected for differential analysis. Finally, *PUS7* (Pseudouridine Synthase 7) as differentially expressed RMGs (DEGs-RMGs) was identified as a diagnostic biomarker candidate and evaluated for its specificity and sensitivity using Receiver Operating Characteristic (ROC) analysis in TCGA and GEO data. The protein expression, mutation, protein interaction networks, correlated genes, related pathways, biological processes, cell components, and molecular functions of *PUS7* were analyzed as well. The upregulation of PUS7 protein in OV was confirmed by the staining images in HPA and tissue arrays. Collectively, the findings of the present study point towards the potential of PUS7 as a diagnostic marker and therapeutic target for ovarian cancer.

## 1. Introduction

Ovarian cancer (OV) is the leading cause of death among gynecologic malignancies in most developed countries [1,2]. It accounts for an estimated 239,000 new cases and 152,000 deaths worldwide annually [3]. The risk of having ovarian cancer during the lifetime of a woman is approximately 1 in 78, and the lifetime chance of dying of ovarian cancer is approximately 1 in 108 [4]. Four out of five OV patients are diagnosed with advanced stage [5], and out of these, only 30% of patients survive more than 5 years [4]. The lack of a practical screening strategy and the asymptomatic characteristic of OV contribute to the late presentation of the disease. Hence, the efficient and early detection of OV is pivotal to improving the survival of ovarian cancer patients.

Post-transcriptional modifications affect RNA stability, localization, structure, splicing, or function [6]. Different RNAs have been detected to contain numerous types of modifications [7,8]. For example, mRNA modifications include N6-methyladenosine (m6A), inosine (I), 5-methylcytosine (m5C), and 5-hydroxymethylcytosine (hm5C). Deregulated RNA modifications are reported to be associated with several pathological processes such as tumorigenesis, cardiovascular diseases, and neurological disorders [9]. RNA modification enzymes have been generally considered important decorations for RNAs [10], and dysregulation and mutation in RNA modification genes are involved in the development of numerous cancers including lung cancer, bladder cancer, leukemia, prostate cancer, breast cancer, etc. [11]. For example, Alpha-Ketoglutarate Dependent Dioxygenase (*FTO*) was deciphered as a prognosticator for lung squamous cell carcinoma and promoted cell proliferation and invasion [12]. Methyltransferase Like 3 (*METTL3*), acting as an oncogene in lung cancer, upregulated *EGFR* and *TAZ* expression and promoted growth, survival, and invasion of human lung cancer cells [13]. NOP2/Sun RNA Methyltransferase 2 (*NSUN2*) was reported to be overexpressed in breast cancer and to be associated with cancer progression [14]. Elongator Acetyltransferase Complex Subunit 3 (*ELP3*), responsible for mcm5s2 modification, has been found to be upregulated in breast cancer and to facilitate cancer cell metastasis [15]. tRNA methyltransferase 9B (*TRM9L/TRMT9B*) has been shown to be downregulated in breast cancer [16]. Similarly, in renal cell carcinomas, G3BP Stress Granule Assembly Factor 1 (*G3BP1*) has been shown to promote tumor progression and metastasis [17]. Taken together, RNA modification genes play pivotal roles in human cancers.

Pseudouridine synthases (PUS) are divided into six families (TruA, TruB, TruD, RsuA, RluA, and Pus10) [18]. PUS7 is the only member of the TruD family that is involved in the modification of tRNAs, at position Tyr35 in pre-tRNA, at position 13 in cytoplasmic tRNA, and at numerous nucleotides in mRNAs. PUS7 is the only pseudouridine synthase to possess a consensus sequence (UGUAR) for substrate recognition [19]. *PUS7* was also reported to be associated with human myeloid malignancies in embryonic stem cells [20]. However, no reports have expounded the role of *PUS7* in OV, so far.

In this study, PUS7 was identified as a novel and potential biomarker for early diagnosis, using transcriptional profiles in the GEO and TCGA databases, ROC, HPA, and Oncomine analyses. Protein–protein interaction (PPI); GSEA pathway; and GO analyses, including the biological process (BP), cell component (CC), and molecular function (MF) terms, were also performed to provide in-depth insights into PUS7.

## 2. Materials and Methods

### 2.1. Data Collection

The RMGs were collected from PubMed according to the keywords “RNA modification”. The transcriptome profiles, including datasets GSE18520 and TCGA, were obtained from GEO (https://www.ncbi.nlm.nih.gov/gds, accessed on 15 October 2019) [21] and UCSC Xena (https://xena.ucsc.edu/, accessed on 16 October 2019) [22], respectively. A total of 53 OV and 10 normal cases were enrolled in GSE18520 (platform: GPL570), and 585 OV and 8 normal cases in TCGA (Affymetrix Human Genome U133 Plus 2.0 Array) were adopted to carry out the following analyses.

### 2.2. Differential Expression Analysis

The GEO2R, an interactive web tool that facilitates users to compare the gene expression between different groups of samples in a GEO dataset, was used to identify the differentially expressed genes (DEGs). The SangerBox was adopted to analyze the TCGA expression profile of ovarian cancer. A *p* value < 0.05 and |log_2_FC| > 1 were used as the cut-off criteria to screen out DEGs. The DEGs of the two datasets were listed in Supplementary Table S1. Subsequently, the RMGs and DEGs that overlapped between GSE18520 and TCGA were selected using Venny 2.1 and were used for further analysis. The analysis of the volcano plot of DEGs in GSE18520 and TCGA, and the heatmaps of DEGs-RMGs in GSE18520 and TCGA were obtained through the SangerBox web tool.

### 2.3. PUS7 Protein Level Analysis of OV Tissues in HPA and Tissue Array

The protein expression of PUS7 was analyzed using HPA data [23]. A tissue chip (HOvaC070PT01) was purchased from SHANGHAI OUTDO BIOTECH CO., LTD. A total of 12 OV samples and 2 healthy ovary samples, and 65 OV samples and 5 healthy ovary samples were retrieved from HPA and tissue array, respectively. The one case with an equivocal staining result was excluded, and the baseline characteristics of the remaining 64 cases of OV tissues in tissue array are described in Supplementary Table S2. The immunohistochemistry (IHC) staining intensity was graded from 0 to 3 (0, negative; 1, weak; 2, moderate; and 3, strong). The staining quantity was graded from 0 to 3 (0, none; 1, <25%; 2, 25–75%; and 3, >75%) according to the percentage of positive cells in the HPA database. The staining quantity was graded from 0 to 4 (0, none; 1, <25%; 2, 25–50%; 3, 50–75%; and 4, >75%) in the tissue assay. The staining scores were calculated by multiplying the staining intensity with the staining quantity.

### 2.4. PUS7 Gene Expression Analysis Using TCGA and GEO Datasets

The *PUS7* expression analysis was carried out using TCGA and GSE119056 expression profiling data. An ROC analysis (the method frequently used for binary assessment) was subsequently performed to evaluate the effectiveness of the expression level of any gene of interest in discriminating between OV and healthy samples. The area under the curve (AUC) value ranged from 0.5 to 1.0, which indicates 50 to 100% discrimination ability.

### 2.5. PUS7 Gene Expression Analysis Using Oncomine Database

The gene expression of *PUS7* was explored using the Oncomine database (https://www.oncomine.org/resource/main.html, accessed on 25 October 2019) [24]. The Oncomine database applies a combination of threshold values (*p*-value) and fold change (FC, tumors vs. controls) with *p* ≤ 0.05 and fold change >1.

### 2.6. Protein–Protein Interaction (PPI) Network Analyses

STRING (https://stringdb.org/, accessed on 22 October 2019) [25] is a database used to predict and analyze functional interactions between proteins and was used to identify the functional protein–protein interactions (PPIs) of *PUS7*. GeneMANIA (http://genemania.org/, accessed on 24 October 2019) [26] was used to identify gene networks embracing *PUS7*.

### 2.7. The Mutation and Correlation Analyses of PUS7

The *PUS7* mutation was performed through cBioPortal (https://www.cbioportal.org/, 27 October 2019) [27]. The Gene Expression Profiling Interactive Analysis (GEPIA) database (http://gepia.cancer-pku.cn/, accessed on 27 October 2020) [28] was employed to analyze the PUS7 correlated genes based on TCGA data.

### 2.8. Pathways and BP, CC, and MF Analyses

Gene set enrichment analysis (GSEA) was carried out to identify potential cellular pathways involved with PUS7. The TCGA-OV dataset was divided into a high (25%) and a low group (75%) based on the PUS7 mRNA expression. Nominal *p*-value < 0.01 and false discovery rate (FDR) q-value < 0.05 were considered significant for enriched gene set analysis. Using 312 genes positively correlated (R > 0.3, *p* < 0.05) with *PUS7* derived from the cBioPortal analysis, the BP, CC, and MF analyses were carried out through the Database for Annotation, Visualization, and Integrated Discovery (DAVID, https://david.ncifcrf.gov/, 19 November 2020) [29] and visualized with bubble diagrams based on *p* values < 0.05.

### 2.9. Statistical Analysis

The statistical analyses were performed using SPSS ver. 26.0. The Student’s *t*-test and the rank-sum test were used to evaluate the difference in PUS7 expression between the OV and normal samples. The ROC curve was constructed using *PUS7* expression profiles in the OV and normal samples by GraphPad Prism 8.0. A *p* value at < 0.05 was taken as a measure of statistically significant difference.

## 3. Results

### 3.1. The Identification of DEGs-RMGs of OV Data in TCGA and GEO

**Figure 1:**
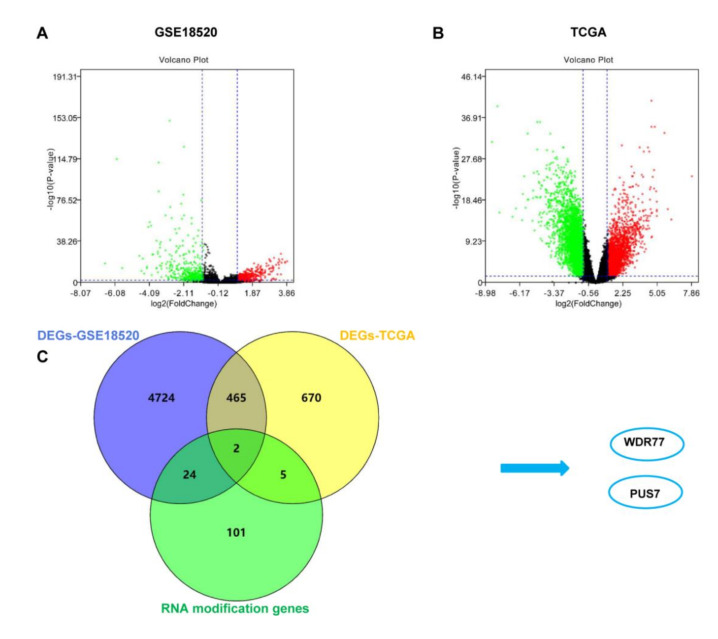
The identification of DEGs-RMGs using OV data in TCGA and GEO. (**A**,**B**) The volcano plot of DEGs between OV and normal samples in GSE18520 and TCGA data. (**C**) *WDR77* and *PUS7* were identified as the overlapping genes of DEGs in both datasets.

**Figure 2:**
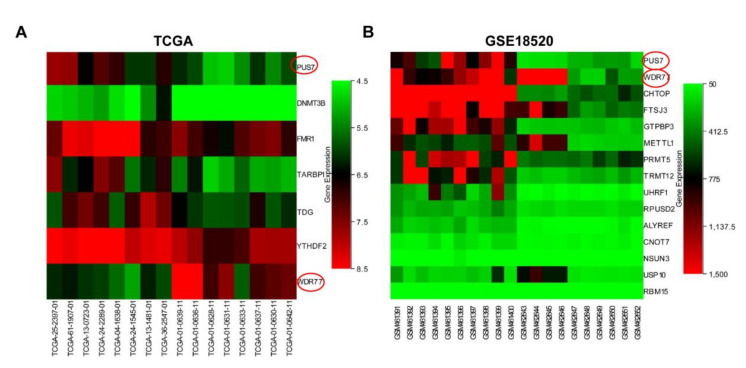
The heatmaps of differentially expressed RMGs. (**A**) The heatmap of the expression profile of overlapping genes of RMGs and DEGs in normal tissues and OV tissues in the TCGA dataset. (**B**) The heatmaps of the expression profile for overlapping genes of RMGs and DEGs in normal tissues and OV tissues in the GSE18520 dataset.

A total of 132 RMGs (Supplementary Table S3) were retrieved from PubMed. TCGA AffyU133a expression profiles and GSE18520 cohorts of ovarian cancer were downloaded from UCSC Xena and the GEO databases, respectively. A total of 1142 and 5215 DEGs (Supplementary Table S1) were obtained in the TCGA dataset and GSE18520 dataset between the OV and normal samples through DEO2R and SangerBox-limma analysis, respectively, and the volcano plots of DEGs are presented in Figure 1A,B. The RMGs and DEGs from the two cohorts were intersected to screen out the overlapping RMGs and DEGs for diagnostic biomarker analysis. As a result, two genes named *WDR77* and *PUS7* were identified as differentially expressed RMGs (Figure 1B). *WDR77* was excluded since it exhibited a contrary expression trend between OV and normal in TCGA and GSE18520 (Figure 2A,B). However, PUS7 showed a consistent high expression in OV rather than normal cases; thus, *PUS7* could be a potential diagnostic biomarker and is subject to further analyses.

### 3.2. Expression Validation and Mutation Analysis for PUS7 in Ovarian Cancer

**Figure 3:**
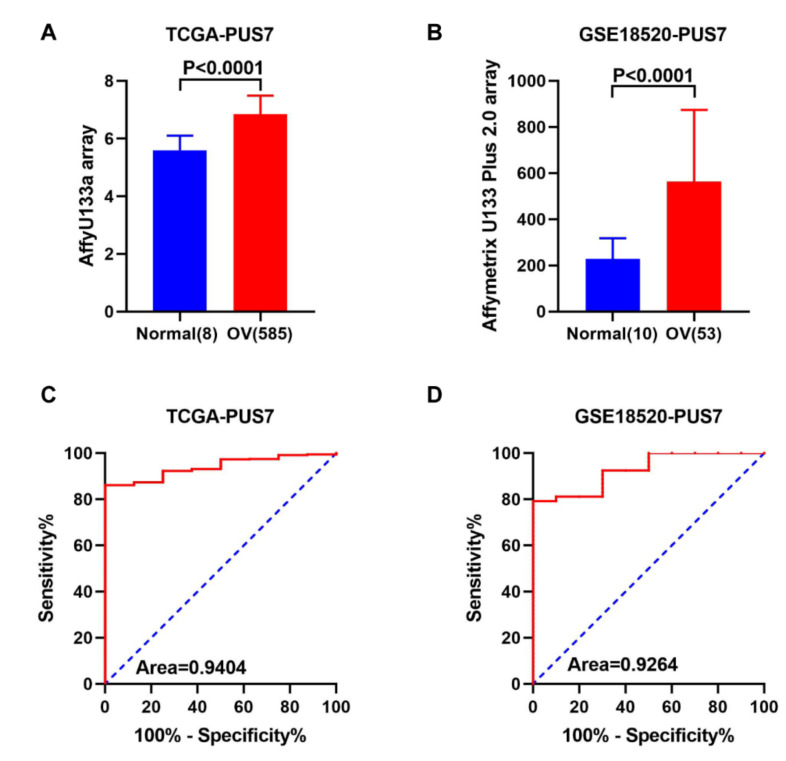
The differential expression analysis and ROC analysis of *PUS7* in OV and normal tissues. (**A**,**B**) The expression analysis of *PUS7* in TCGA and GSE18520 cohorts, respectively. (**C**,**D**) The ROC analysis of PUS7 between OV and normal samples in TCGA and GSE18520 cohorts. AUC is plotted as sensitivity% vs. 100-specificity%. A *p* < 0.05 was considered a significant difference.

**Figure 4:**
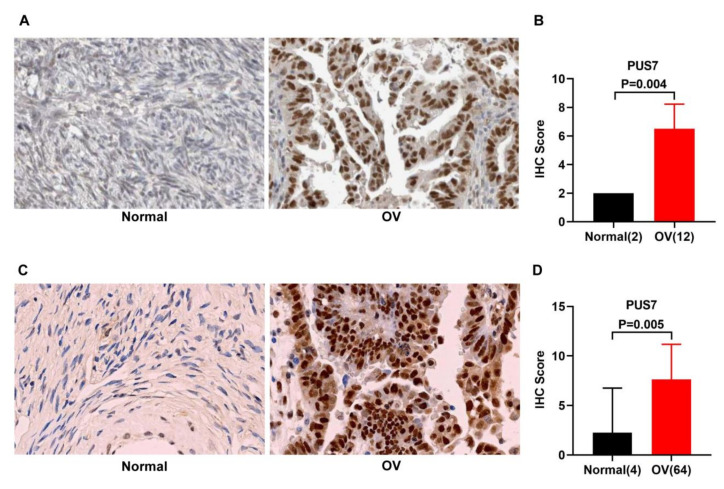
PUS7 protein expression was significantly higher in OV tissues than normal tissues. (**A**) Representative IHC images of PUS7 in normal (**left**) and OV (**right**) tissues in HPA. (**B**) Statistical analysis of the protein expression of PUS7 according to the staining scores of OV and normal tissues. (**C**) Representative IHC images of PUS7 in normal (**left**) and OV (**right**) tissues according to tissue microarray. (**D**) Statistical analysis of the protein expression of PUS7 according to the staining scores of OV and normal tissues. *p* < 0.05 was considered significant.

To validate the overexpression of *PUS7* in OV rather than normal samples, an Oncomine analysis was performed on ovarian cancer with different pathological types, and found that the *PUS7* expression is highly elevated in OV samples with fold change >1 and *p* < 0.05 (as presented in Figure 3A,B and Table 1). Moreover, Figure 3C,D displays the corresponding ROC curve of *PUS7* in the TCGA and GSE18520 datasets, indicating the remarkable potential of *PUS7* to discriminate OVs from normal tissue. The IHC analytic results showed the overexpression of PUS7 at the protein level (Figure 4A,B). To further explore the overexpression of PUS7 at the protein level in OV samples, a tissue array was performed. Typical staining images in the tissue array are exhibited in Figure 4C, confirming the protein upregulation of PUS7 in OV tissues (Figure 4D). Since mutations in RNA modification genes have been reported to be associated with several types of human cancers, the mutation analysis of PUS7 was performed in cBioPortal, demonstrating the fusion of PUS7 with SRSF Protein Kinase 2 (SRPK2) in serous ovarian cancer (Table 2).

### 3.3. The Interaction Network of PUS7

**Figure 5:**
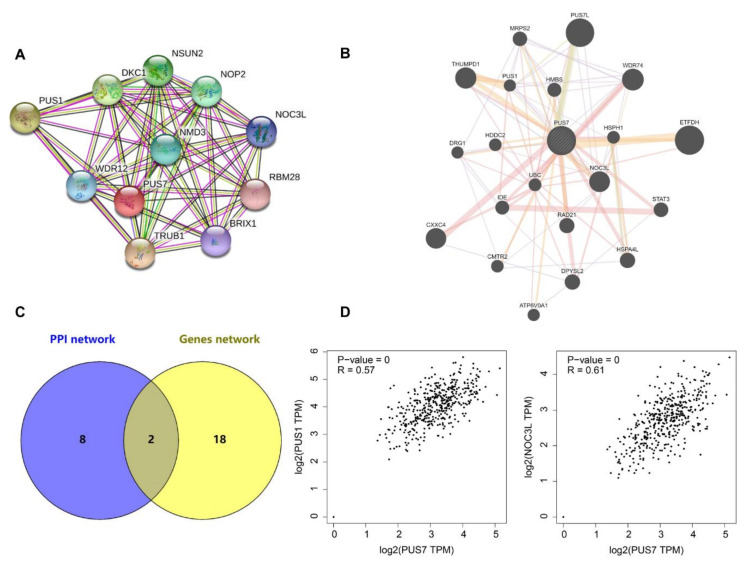
The interaction network and correlation of *PUS7*. (**A**) The protein interaction network of PUS7. Ten proteins including NSUN2, NOP2, NOC3L, RBM28, BRIX1, TRUB1, WDR12, PUS1, DKC1, and NMD3 physically/functionally interact with PUS7. (**B**) Twenty genes named *ETFDH*, *WDR74, THUMPD1, NOC3L, CXXC4, DPYSL2, HSPA4L, RAD21, STAT3, MRPS2, HMBS, IDE, UBC, HSPH1, HDDC2, CMTR2, ATP6V0A1,* and *DRG1* have physical interactions or genetic interactions, share protein domains with *PUS7*, or co-express or co-localize with *PUS7*. (**C**) Two genes were shared by the two networks. (**D**) The correlation analysis of *PUS7* with *PUS1* and *NOC3L*. R > 0.5 plus *p* < 0.05 was regarded as a significant correlation.

To explore the PPI and gene networks of PUS7 and its partner, an analysis using the String and GeneMANIA tools was performed. The PPI analysis results showed that a total of 10 proteins including NSUN2, NOP2, NOC3L, RBM28, BRIX1, TRUB1, WDR12, PUS1, DKC1, and NMD3 have interactions with PUS7 (Figure 5A). In the GeneMANIA analysis, a total of 20 genes named *ETFDH*, *WDR74*, *THUMPD1*, *NOC3L*, *CXXC4*, *DPYSL2*, *HSPA4L*, *RAD21*, *STAT3*, *MRPS2*, *HMBS*, *IDE*, *UBC*, *HSPH1*, *HDDC2*, *CMTR2*, *ATP6V0A1*, and *DRG1* were demonstrated to have physical interactions or genetic interactions or to share protein domains with *PUS7* or were co-expressed or co-located with *PUS7* (Figure 5B). The shared genes of the above two analyses are *PUS1* and *NOC3L* (Figure 5C), where *PUS1* was co-expressed with *PUS7* [30,31] and *NOC3L* physically interacted with *PUS7* [31,32,33] in GeneMANIA, both of which were known to interact with PUS7, according to the String results. In addition, GEPIA analysis showed that the expression of *PUS7* is significantly correlated with *PUS1* (R = 0.57, *p*-value = 0) and *NOC3L* (R = 0.61, *p*-value = 0) (Figure 5D).

### 3.4. The Pathway Enrichment Analysis of PUS7 in Ovarian Cancer

**Figure 6:**
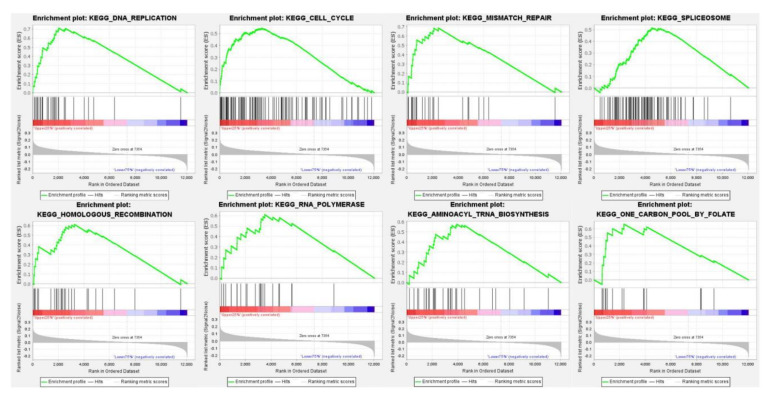
The pathway enrichment analysis of PUS7 in ovarian cancer. GSEA pathway analysis using TCGA ovarian cancer data, which was separated to a high (top25%) *PUS7* group and a low (down75%) *PUS7* group. Eight top pathways in which *PUS7* participates were DNA replication, the cell cycle, mismatch repair, spliceosomes, homologous recombination, RNA polymerase, aminoacyl, tRNA biosynthesis, and one carbon pool by folate in ovarian cancer.

To investigate the pathways that *PUS7* may be involved in or may regulate in ovarian cancer, a GSEA pathway analysis was performed using TCGA data, which was separated into a high (top 25%) *PUS7* group and a low (down 75%) *PUS7* group. The top eight pathways in which PUS7 participates are DNA replication, the cell cycle, mismatch repair, spliceosomes, homologous recombination, RNA polymerase, aminoacyl tRNA biosynthesis, and one carbon pool by folate in ovarian cancer (Figure 6). Among the eight pathways, the top two pathways are DNA replication and the cell cycle, both of which are linked to ovarian cancer cell proliferation. These results may imply that the overexpression of *PUS7* in ovarian cancer might promote ovarian cancer proliferation via regulation of DNA replication and the cell cycle.

### 3.5. Gene Ontology (GO) Analyses of PUS7 in Ovarian Cancer

**Figure 7:**
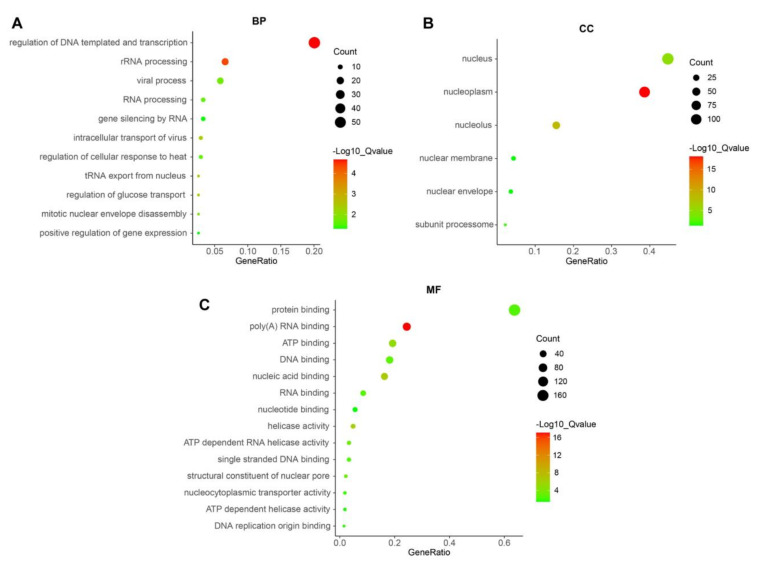
The GO analyses of PUS7 in ovarian cancer. The bubble diagrams were analyzed using PUS7-related genes and exhibited the biological processes (**A**), cellular components, (**B**) and molecular functions (**C**) of PUS7.

To further clarify the GO terms of BP (biological processes), CC (cellular components) and MF (molecular functions) of *PUS7*, a total of 312 genes (Supplementary Table S4) positively related to *PUS7* (R > 0.3, *p* < 0.0001) according to the TCGA ovarian cancer data through the cBioPortal database were subjected to DAVID analysis. The results showed that biological processes in which *PUS7* mainly participates include the regulation of DNA templates and transcription, rRNA processing, tRNA export from nuclei, the regulation of glucose transport, the intracellular transport of viruses, mitotic nuclear envelope disassembly, viral processes, RNA processing, the regulation of cellular response to heat, gene silencing by RNA, and the positive regulation of gene expression (Figure 7A). The cellular components affected by *PUS7* include the nucleoplasm, nucleolus, nucleus, small subunit processomes, nuclear envelope, and nuclear membrane (Figure 7B). The molecular functions of *PUS7* include poly(A) RNA binding, nucleic acid binding, helicase activity, ATP binding, ATP-dependent RNA helicase activity, structural constituents of a nuclear pore, DNA binding, RNA binding, protein binding, single-stranded DNA binding, nucleocytoplasmic transporter activity, DNA replication origin binding, ATP-dependent helicase activity, and nucleotide binding (Figure 7C).

## 4. Discussion

It was estimated that there were 22,530 new cases and 13,980 deaths due to ovarian cancer in the United States in 2019 [34]. Ovarian cancers are often diagnosed late, when the disease has progressed to advanced stages. Hence, an efficient and reliable diagnostic marker is very necessary to facilitate clinical diagnosis and to prolong the survival time for OV. RNA modifications are reported to play vital roles in human diseases, including cancer. For example, m6A, a new star of RNA modifications, is associated with tumorigenesis, tumor proliferation and differentiation and functions as oncogenes or anti-oncogenes in malignant tumors [35]. For example, m6A plays a pivotal role in ovarian cancer progression [36]. Recent advances in human Mendelian diseases have brought focus to human PUS genes as a type of RMG in clinical medicine [37]. PUS7-mediated pseudouridylation could “activate” a class of tRNA-derived small RNAs to regulate protein synthesis and stem cell fate [20]. Additionally, PUS7 is also reported to be a potential biomarker for glioma [38].

In this study, we investigated dysregulated RMGs in ovarian cancer and identified PUS7 as a novel potential biomarker for the diagnosis of OV. ROC analysis acting as an efficient method has been commonly used to determine the accuracy and specificity of medical imaging techniques and non-imaging diagnostic tests in various settings involving disease screening, prognosis, diagnosis, staging, and treatment [39]. Herein, ROC analysis aimed at discriminating cancer from normal tissue was performed to evaluate the sensitivity and specificity of PUS7 in GEO and TCGA data. AUC is a global measure of the ability of a test to discriminate whether a specific condition is present [40]. In this study, an AUC score over 0.9 in an ROC analysis was obtained, suggesting the potent discriminating potency of PUS7 (AUC = 0.9404, *p* < 0.0001) in ovarian cancer. In addition to PUS7 upregulation in the TCGA and GEO datasets, the Oncomine database analysis and IHC results further validated the promising diagnostic role of PUS7 in OV.

PUS7 has never been reported in ovarian cancer. To rationalize the vital role of PUS7 in OV, we explored the proteins interacting with PUS7, which may partially help explain PUS7 function in tumor diagnosis, tumorigenesis, and development. The PPI and gene network analyses identified PUS7 interacting partners, including NOC3L and PUS1, which are also not reported in ovarian cancer, although several reports have revealed that NOC3L regulates the proliferation and tumorigenesis of gastric cancer [41], and NOC3L is associated with an increased risk of gastric cancer in the Chinese Han population [42]. For PUS1, previous reports demonstrated that it is related to sideroblastic anemia [43], and no association of PUS1 with cancer was ever shown, suggesting the novelty of the protein interaction. To further explore the signaling pathway of *PUS7* in ovarian cancer, the GSEA pathways analysis demonstrated that DNA replication and the cell cycle are the top two pathways that *PUS7* regulated. These results point towards the role of *PUS7* in ovarian cancer proliferation via regulation of DNA replication and the cell cycle. However, this hypothesis needs further experiments to be validated.

## 5. Conclusions

In conclusion, the findings of the present study revealed PUS7 as a novel and prospective biomarker at the RNA and protein levels for ovarian cancer. Further analysis indicated that PUS7 may interact with NOC3L and PUS1 to regulate ovarian cancer proliferation via modulation of DNA replication and the cell cycle.

## Figures and Tables

**Table 1:** The comparison analysis of *PUS7* in ovarian cancer and normal tissue in different cohorts (Oncomine).

Dataset	Tumor (Cases)	Normal (Cases)	Fold Change	*t*-Test	*p*-Value
Lu Ovarian	Ovarian Serous Adenocarcinoma (20)	Ovarian Surface Epithelium (5)	1.913	9.134	2.28 × 10^−9^
Lu Ovarian	Ovarian Endometrioid Adenocarcinoma (9)	Ovarian Surface Epithelium (5)	1.808	5.846	0.0000904
Lu Ovarian	Ovarian Mucinous Adenocarcinoma (9)	Ovarian Surface Epithelium (5)	1.405	4.275	0.000692
Lu Ovarian	Ovarian Clear Cell Adenocarcinoma (7)	Ovarian Surface Epithelium (5)	1.457	2.64	0.017
Hendrix Ovarian	Ovarian Mucinous Adenocarcinoma (13)	Ovary (4)	1.216	4.26	0.003
Hendrix Ovarian	Ovarian Clear Cell Adenocarcinoma (8)	Ovary (4)	1.275	4.44	0.000934
Hendrix Ovarian	Ovarian Endometrioid Adenocarcinoma (37)	Ovary (4)	1.299	6.012	0.00098
Hendrix Ovarian	Ovarian Serous Adenocarcinoma (37)	Ovary (4)	1.301	6.304	0.001
Yoshihara Ovarian	Ovarian Serous Adenocarcinoma (43)	Peritoneum (10)	1.537	3.171	0.003

**Table 2:** The mutation distribution of PUS7 in ovarian cancer according to cBioPortal.

Cancer Type	Sample ID	Fusion Partner	Copy	Mutation in Sample
Serious Ovarian Cancer	TCGA-24-1469-01	Fusion, SRPK2-PUS7	ShallowDel	223
Serious Ovarian Cancer	TCGA-31-1953-01	Fusion, SRPK2-PUS7	Gain	56
Serious Ovarian Cancer	TCGA-61-1740-01	Fusion, SRPK2-PUS7	Gain	183

SRPK 2. SRSF Protein Kinase 2; PUS7: Pseudouridine Synthase 7.

## Data Availability

Not applicable.

## Supplementary Materials

The following are available online at https://www.mdpi.com/article/10.3390/biology10111130/s1, Table S1: The differentially expressed genes in ovarian cancer samples compared with normal tissue according to TCGA and GSE18520 data. Table S2: The baseline characteristics of ovarian cancer samples in a tissue array. Table S3: RNA modification-related genes. Table S4: The genes correlated to PUS7.

## References

[1] Myriam K., Alexandra L., Jean-Yves S., Catherine G. (2018). Ovarian Cancer: A Heterogeneous Disease. Pathobiology.

[2] Giusti I., Bianchi S., Nottola S.A., Macchiarelli G., Dolo V. (2019). Clinical electron microscopy in the study of human ovarian tissues. Euromediterranean Biomed. J..

[3] Brett M.R., Jennifer B.P., Thomas A.S. (2017). Epidemiology of ovarian cancer: A review. Cancer Biol. Med..

[4] Rebecca A., Alba M., Tomasz S., Michael J.B. (2019). Biomarkers in ovarian cancer: To be or not to be. Cancer.

[5] Lindsey A.T., Britton T., Carol E.D., Kimberly D.M., Goli S., Carolyn D.R., Mia M.G., Ahmedin J., Rebecca L.S. (2018). Ovarian Cancer Statistics, 2018. CA Cancer J. Clin..

[6] Roundtree I.A., Evans M.E., Pan T., He C. (2017). Dynamic RNA Modifications in Gene Expression Regulation. Cell.

[7] Machnicka M.A., Olchowik A., Grosjean H., Bujnicki J.M. (2014). Distribution and frequencies of post-transcriptional modifications in tRNAs. RNA Biol..

[8] Cantara W.A., Crain P.F., Rozenski J., McCloskey J.A., Harris K.A., Zhang X., Vendeix F.A.P., Fabris D., Agris P.F. (2011). The RNA Modification Database, RNAMDB: 2011 update. Nucleic Acids Res..

[9] Carell T., Brandmayr C., Hienzsch A., Muller M., Pearson D., Reiter V., Thoma I., Thumbs P., Wagner M. (2012). Structure and function of noncanonical nucleobases. Angew. Chem. Int. Ed. Engl..

[10] Karijolich J., Yu Y.T. (2010). Spliceosomal snRNA modifications and their function. RNA Biol..

[11] Nicky J., Julia T., Martin A.S., Nicole S., John S.M., Eva M.N. (2017). The RNA modification landscape in human disease. RNA.

[12] Liu J., Ren D., Du Z., Wang H., Zhang H., Jin Y. (2018). m6A demethylase FTO facilitates tumor progression in lung squamous cell carcinoma by regulating MZF1 expression. Biochem. Biophys. Res. Commun..

[13] Lin S., Junho C., Du P., Robinson T., Richard I.G. (2016). The m6A methyltransferase METTL3 promotes translation in human Cancer cells. Mol. Cell.

[14] Yi J., Gao R., Chen Y., Yang Z., Han P., Zhang H., Dou Y., Liu W., Wang W., Du G. (2016). Overexpression of NSUN2 by DNA hypomethylation is associated with metastatic progression in human breast cancer. Oncotarget.

[15] Sylvain D., Francesca R., Lars T., Zhou Z., Lukas H., Martin T., Kateryna S., Iva K., Alexandra F., Hadrien D. (2016). Elp3 links tRNA modification to IRES-dependent translation of LEF1 to sustain metastasis in breast cancer. J. Exp. Med..

[16] Begley U., Sosa M.S., Avivar-Valderas A., Patil A., Endres L., Estrada Y., Chan C.T., Su D., Dedon P.C., Aguirre-Ghiso J.A. (2013). A human tRNA methyltransferase 9-like protein prevents tumour growth by regulating LIN9 and HIF1-α. EMBO Mol. Med..

[17] Wang Y., Fu D., Chen Y., Su J., Wang Y., Li X., Zhai W., Niu Y., Yue D., Geng H. (2018). G3BP1 promotes tumor progression and metastasis through IL-6/G3BP1/STAT3 signaling axis in renal cell carcinomas. Cell Death Dis..

[18] Tomoko H., Adrian R.F. (2006). Pseudouridine synthases. Chem. Biol..

[19] Rintala-Dempsey A.C., Kothe U. (2017). Eukaryotic stand-alone pseudouridine synthases-RNA modifying enzymes and emerging regulators of gene expression?. RNA Biol..

[20] Guzzi N., Cieśla M., Ngoc P.C.T., Lang S., Arora S., Dimitriou M., Pimková K., Sommarin M.N.E., Munita R., Lubas M. (2018). Pseudouridylation of tRNA-Derived Fragments Steers Translational Control in Stem Cells. Cell.

[21] Ron E., Michael D., Alex E.L. (2002). Gene Expression Omnibus: NCBI gene expression and hybridization array data repository. Nucleic Acids Res..

[22] Goldman M., Craft B., Swatloski T., Cline M., Morozova O., Diekhans M., David Haussler D., Zhu J. (2015). The UCSC cancer genomics browser: Update 2015. Nucleic Acids Res..

[23] Pontén F., Jirström K., Uhlen M. (2008). The Human Protein Atlas-a tool for pathology. J. Pathol..

[24] Rhodes D.R., Yu J., Shanker K., Deshpande N., Varambally R., Ghosh D., Barrette T., Pandey A., Chinnaiyan A.M. (2004). ONCOMINE: A cancer microarray database and integrated data-mining platform. Neoplasia.

[25] Snel B., Lehmann G., Bork P., Huynen M.A. (2000). STRING: A web-server to retrieve and display the repeatedly occurring neighbourhood of a gene. Nucleic Acids Res..

[26] Sara M., Debajyoti R., David W.-F., Chris G., Quaid M. (2008). GeneMANIA: A real-time multiple association network integration algorithm for predicting gene function. Genome Biol..

[27] Cerami E., Gao J., Dogrusoz U., Gross B.E., Sumer S.O., Aksoy B.A., Jacobsen A., Byrne C.J., Heuer M.L., Larsson E. (2012). The cBio cancer genomics portal: An open platform for exploring multidimensional cancer genomics data. Cancer Discov..

[28] Tang Z., Li C., Kang B., Gao G., Li C., Zhang Z. (2017). GEPIA: A web server for cancer and normal gene expression profiling and interactive analyses. Nucleic Acids Res..

[29] Huang D.W., Sherman B.T., Lempicki R.A. (2009). Systematic and integrative analysis of large gene lists using DAVID bioinformatics resources. Nat. Protoc..

[30] Innocenti F., Cooper G.M., Stanaway I.B., Gamazon E.R., Smith J.D., Mirkov S., Ramirez J., Liu W., Lin Y.S., Moloney C. (2011). Identification, replication, and functional fine-mapping of expression quantitative trait loci in primary human liver tissue. PLoS Genet..

[31] Denis A.S., Michael M., Eunice S., Richard S.S., Vivian G.C. (2009). Genetic analysis of radiation-induced changes in human gene expression. Nature.

[32] Wang I.X., Ramrattan G., Cheung V.G. (2015). Genetic variation in insulin-induced kinase signaling. Mol. Syst. Biol..

[33] Wan C., Borgeson B., Phanse S., Tu F., Drew K., Clark G., Xiong X., Kagan O., Kwan J., Bezginov A. (2015). Panorama of ancient metazoan macromolecular complexes. Nature.

[34] Siegel R.L., Miller K.D., Jemal A. (2019). Cancer statistics. CA Cancer J. Clin..

[35] Chen X., Zhang J., Zhu J. (2019). The role of m6A RNA methylation in human cancer. Mol. Cancer.

[36] Liu T., Wei Q., Jin J., Luo Q., Liu Y., Yang Y., Cheng C., Li L., Pi J., Si Y. (2020). The m6A reader YTHDF1 promotes ovarian cancer progression via augmenting EIF3C translation. Nucleic Acids Res..

[37] Shaheen R., Tasak M., Maddirevula S., Abdel-Salam G., Sayed I., Alazami A., Al-Sheddi T., Eman Alobeid E., Phizicky E., Alkuraya F. (2019). PUS7 Mutations Impair Pseudouridylation in Humans and Cause Intellectual Disability and Microcephaly. Hum. Genet..

[38] Hu G., Wang R., Wei B., Wang L., Yang Q., Kong D., Du C. (2020). Prognostic Markers Identification in Glioma by Gene Expression Profile Analysis. J. Comput. Biol..

[39] Obuchowski N.A., Bullen J.A. (2018). Receiver operating characteristic (ROC) curves: Review of methods with applications in diagnostic medicine. Phys. Med. Biol..

[40] Zhe H., Jane C., Dawn T. (2017). What is an ROC curve?. Emerg. Med. J..

[41] Yan C., Zhu M., Ding Y., Yang M., Wang M., Li G., Ren C., Huang T., Yang W., He B. (2020). Meta-analysis of genome-wide association studies and functional assays decipher susceptibility genes for gastric cancer in Chinese populations. Gut.

[42] Yuan L., Jin T., Yin J., Du X., Wang Q., Dong R., Wang S., Cui Y., Chen C., Lu G. (2012). Polymorphisms of tumor-related genes IL-10, PSCA, MTRR and NOC3L are associated with the risk of gastric cancer in the Chinese Han population. Cancer Epidemiol..

[43] Tesarova M., Vondrackova A., Stufkova H., Veprekova L., Stranecky V., Berankova K., Hansikova H., Magner M., Galoova N., Honzik T. (2019). Sideroblastic anemia associated with multisystem mitochondrial disorders. Pediatr. Blood Cancer.

