# Supplementary material for: The Identification of RNA Modification Gene PUS7 as a Potential Biomarker of Ovarian Cancer
Source: Biology (Basel). 2021 Nov 3;10(11):1130. doi: 10.3390/biology10111130 (PMC8615213; doi:10.3390/biology10111130)
Supplement: Supplementary file 1 [file biology-10-01130-s001.zip › Supplementary files/biology-1421131 supplementary English.pdf]

Table S2. The baseline characteristics of ovarian cancer samples in a tissue array.

Variable	N (%)	Median (IHC score)	*p*-value
**Age**			
≤60	44 (68.7%)	8	0.861
＞60	18 (28.1%)	8	
NA	2 (3.2%)		
**Lymphatic invasion**			
No	60 (93.8%)	8	0.165
Yes	4 (6.2%)	5.5	
**Pathological type**			
Serous ovarian cancer	38 (59.3%)	8	0.650
Mucinous cancer	7 (10.9%)	9	
Endometrial cancer	4 (6.3%)	10	
Ovarian clear cell cancer	4 (6.3%)	9.5	
Other	11 (17.2%)	7	
**Clinical stage**			
I, I–II, II	35 (54.7%)	8	0.972
II–III, III	19 (29.7%)	8	
NA	10 (15.6%)		
**Tumor location**			
Bilateral ovaries	14 (21.9%)	8	0.700
Unilateral ovarian	47 (73.4%)	8	
NA	3 (4.7%)		

NA: Clinical information is not available.
